# Adrenocortical carcinoma (ACC): diagnosis, prognosis, and treatment

**DOI:** 10.3389/fcell.2015.00045

**Published:** 2015-07-03

**Authors:** Rossella Libé

**Affiliations:** Department of Endocrinology, French Network for Adrenal Cancer, Cochin HospitalParis, France

**Keywords:** adrenocortical carcinoma (ACC), ENS@T staging, prognosis, mitotane, target therapy

## Abstract

Adrenocortical carticnoma (ACC) is a rare malignancy with an incidence of 0.7–2.0 cases/million habitants/year. The diagnosis of malignancy relies on careful investigations of clinical, biological, and imaging features before surgery and pathological examination after tumor removal. Most patients present with steroid hormone excess or abdominal mass effects, but 15% of patients with ACC is initially diagnosed incidentally. After the diagnosis, in order to assess the ACC prognosis and establish an adequate basis for treatment decisions different tools are proposed. The stage classification proposed by the European Network for the Study of Adrenal Tumors (ENSAT) is recommended. Pathology reports define the Weiss score, the resection status and the proliferative index, including the mitotic count and the Ki67 index. As far as the treatment is concerned, in case of tumor limited to the adrenal gland, the complete resection of the tumor is the first option. Most patients benefit from adjuvant mitotane treatment. In metastatic disease, mitotane is the cornerstone of initial treatment, and cytotoxic drugs should be added in case of progression. Recently, the First International Randomized (FIRM-ACT) Trial in metastatic ACC reported the association between mitotane and etoposide/doxorubicin/cisplatin (EDP) as the new standard in first line treatment of ACC. In last years, new targeted therapies, including the IGF-1 receptor inhibitors, have been investigated, but their efficacy remains limited. Thus, new treatment concepts are urgently needed. The ongoing “omic approaches” and next-generation sequencing will improve our understanding of the pathogenesis and hopefully will lead to better therapies.

## Introduction

Adrenocortical carticnoma (ACC) is a rare malignancy with an incidence of 0.7–2.0 cases/million habitants/year. It occurs at any age, with two peak incidence: the first one in the first decade and the second one between 40 and 50 years. Women are most frequently affected (55–60%) (Kebebew et al., 2006).

## Molecular oncogenesis and the epigenetic aspects

In the past, progress in identifying genes involved in ACC came mainly from the study of familial diseases (Else et al., 2014). ACC were frequently associated to the Li–Fraumeni syndrome, due to germline TP53 mutations and the Beckwith–Wiedemann syndrome, due to alterations of the insulin-like growth factor IGF2. At somatic level, inactivating mutations of TP53 and activating mutations of the proto-oncogene β-catenin (CTNNB1) were the most frequent mutations identified in ACC. Recently, thanks to genomic approaches, including exome sequencing, not only TP53 and CTNNB1 were confirmed as implicated in ACC tumorigenesis, but also ZNRF3 (Zinc and ring finger protein 3) was the most frequently altered gene (21%) (Assié et al., 2014a). Interestingly, ZNRF, as CTNNBI, belong to the WNT pathway and it seems that the mutations in the two genes are mutually exclusive.

Moreover, by comparative genomic hybridization (CGH), chromosomal gains at 5, 7, 12, 16, 19, and 20 and losses at 13 and 22 were observed in ACC. Concerning the epigenetic changes, a specific CpG island methylator phenotype was identified in ACC associated to the hypermethylation of the promoters of specific genes as H19, PLAGL1, G0S2, and NDRG2. In addition, some studies identified a significant up-regulation of miR-483 associated to a downrefulation of miR-195 and miR-335 in ACC (Assié et al., 2014b).

## Diagnosis

### Endocrine work-up

The diagnosis of malignancy relies on careful investigations of clinical, biological, and imaging features before surgery and pathological examination after tumor removal. Most patients (40–60%) present steroid hormone excess (glucocorticoids, mineralocorticoids, androgens) or abdominal mass effects (30%), but 15–20% of patients with ACC are initially diagnosed incidentally (Else et al., 2014).

The European Network for the Study of Adrenal Tumors (ENS@T) suggests a pre-operative hormonal workup for suspected ACC (www.ensat.org). In particular, the assessment of basal cortisol, ACTH, dehydroepiandrostenedione sulfate, 17-hydroxyprogesterone, testosterone, androstenedione, and estradiol as well as a dexamethasone suppression test and urinary free cortisol are recommended. In the last years, it seems more evident that some ACC, previously considered as non-secreting, in fact can secrete some urine steroid metabolites and recently urine steroid metobolomic analysis have been introduced in routine use (Arlt et al., 2012).

### Imaging

Traditional and functional imagings are able to diagnose correctly an adrenal mass as ACC in most of the cases. The risk for ACC increases with tumor size, with the index of suspicion increasing for tumors >4 cm (sensitivity, 97%; specificity, 52%) and >6 cm (sensitivity, 91%; specificity, 80%) (Sturgeon et al., 2006). Unfortunately, masses from 1 to 4 cm in diameter are diagnostically challenging. Generally, most of the ACC are large, heterogeneous with irregular margins. Necrosis, hemorrhage or calcification can be associated.

Currently, no single imaging method can characterize a localized adrenal mass as ACC. Regarding traditional imaging, abdominal computed tomography (CT) scan is mandatory in suspicion of ACC: many studies have established a threshold of ≤10 Hounsfield Unit (HU) in unenhanced CT for the diagnosis of benign lesion. When the basal density is >10 UH, the contrast media washout is helpful to discriminate the benign adrenal lesions from the ACC. An absolute washout >50% suggests a benign adrenal lesion. As well as CT scan is fundamental to define the disease staging (Ilias et al., 2007; Zhang et al., 2010; Young, 2011), all patients with ACC must perform a chest CT scan in order to detect pulmonary metastases before surgery.

The state of art of the Magnetic Resonance Imaging (MRI) is less known. In case of suspicion of ACC, when the CT scan cannot perfectly characterize the adrenal lesion, three major characteristics of MRI are helpful in the ACC diagnosis: the presence of isointense to hypointense signal on T1-weighted images, a hyperintense signal on T2-weighted images and an heterogeneous signal drop on chemical shift (Elsayes et al., 2004; Bharwani et al., 2011).

Regarding functional imaging, ACC showed high 18F-fluorodeoxyglucose (FDG) uptake (Boland et al., 2011; Deandreis et al., 2014) with a cut-off value > 1.45 for adrenal to liver maximum standardized uptake value (SUV), as reported in a series of 77 patients with surgical proven diagnosis of adrenal adenoma or ACC (Groussin et al., 2009). As the chest and abdominal CT scan, FDG-PET is important for disease staging and prognosis (Leboulleux et al., 2006), but its routine use still needs validation.

In recent years, a new tracer, the metomidate ([^11^C]MTO) can be useful to prove the adrenocortical origin because it specifically binds to adrenocortical CYP11B enzymes, which catalyze the final steps of steroid synthesis. In a study of 11 patients, ACC showed a higher tracer uptake at [^11^C]MTO-PET compared to normal adrenal gland and liver (Hahner et al., 2008).

### Pathology

The pathological assessment is the key to the final diagnosis of ACC, but it remains challenging. First, as the ACC can be non-secreting tumor, the adrenocortical origin of the mass must be established. The determination of steroidogenic factor 1 (SF-1) expression has proved as the most valid marker (Duregon et al., 2009; Sbiera et al., 2009). Second, multiple parameters (macroscopic and microscopic) have to be evaluated in order to discriminate benign from malignant tumor.

Macroscopy revelead that ACC are usually large, heterogeneous, with a surface ranges from brown to orange to yellow depending on the lipid content of their cells. Necrosis is almost always present. Importantly, the presence of a tumoral invasion at different levels, as the tumor capsule, the extra-adrenal soft tissue or direct invasion of lymphatic channels, blood vessels are the key features of ACC.

Microscopically, the Weiss score is still the best validated score. It is composed of nine items (three concerning the architecture, three the nucleus, and three the presence of any type of invasion) and the presence of one item scores 1. The sum of the positive items defines the final score. It is established that a Weiss score ≥ 3 define an ACC, whereas scores between 0 and 2 defines the adrenal adenoma, even if sometimes a Weiss score of 2 can be suspicious (Weiss, 1984).

The major problem is the reproducibility of this score and in particular the inter-individual reproducibility. Recently, the practice of the Weiss score through virtual microscopy has been improved by the 12 pathologists of the French network for Adrenal Cancer COMETE (Tissier et al., 2012).

Proliferation index, as Ki67 immunomarker or mitotic count, can help to define the diagnosis and prognosis of ACC. It is well-established that ACC generally showed a Ki67 ≥ 5%. Recent studies have been demonstrated that Ki67 is a powerful prognostic marker in both localized and metastatic ACC to guide treatment decision (Berruti et al., 2010; Libé et al., 2014; Beuschlein et al., 2015). Moreover, a mitotic count >20 mitoses/50 HPF defines a “high grade ACC” with a worst prognosis compared to “low grade ACC” with ≤ 20 mitoses/50 HPF (Miller et al., 2010).

## Staging

Tumor staging is a widely used tool to assess prognosis in patients with cancer. For ACC, the tumor–node–metastasis (TNM) classification proposed by ENS@T (Table 1) is recommended (Fassnacht et al., 2009). This staging system, defines stage I and stage II as strictly localized tumors with a size of ≤5 or >5 cm, respectively. Stage III ACC are characterized by infiltration in surrounding tissue, positive regional lymph nodes or a tumor thrombus in the vena cava and/or renal vein, whereas stage IV is defined by the presence of distant metastasis. The high prognostic potential of the ENS@T staging system has been established in the large cohort of the German ACC registry (Beuschlein et al., 2015) and has been confirmed in the independent SEER cohort (Lughezzani et al., 2010) which demonstrates its superiority to the staging system published by the Union Internationale Contre Le Cancer (UICC).

**Table 1:** **ENS@T classification**.

**ENS@T stage**	
I	T1, N0, M0
II	T2, N0, M0
III	T3–T4, N1
IV	T1–T4, N0–N1, M1

*T1, tumor ≤ 5 cm; T2, tumor > 5 cm; T3, histologically proven tumor invasion of surrounding tissue; T4, tumor invasion of adjacent organs or venous tumor thrombus in vena cava or renal vein. Venous tumor thrombus is only a criterion in the ENSAT classification*.

*N0, negative lymph nodes; N1, positive lymph nodes*.

*M0, absence of distant metastases; M1, presence of distant metastases*.

## Prognosis

Three major criteria are mandatory in order to define the disease free survival for the localized ACC (stage I, II, and some III) and the overall survival for stage IV ACC: (1) staging; (2) resection status “R”; (3) Grading (proliferation index, as Ki67% and mitotic count).

### Staging

As mentioned above, staging is mandatory to assess prognosis. Five-year stage-dependent survival is 66–82% for stage I, 58–64% for stage II, 24–50% for stage III, and 0–17% for stage IV, according to different series (Icard et al., 2001; Fassnacht et al., 2009; Lughezzani et al., 2010; Kerkhofs et al., 2013).

### Resection status “R”

In localized ACC, surgery is the single most important intervention and the complete resection (R0) correlates with a better prognosis (Bilimoria et al., 2008). In fact, an incomplete microscopic resection (R1), an incomplete macroscopic resection (R2) or unknown resection (Rx) are associated with the worst overall survival of 20 and 15%, respectively (Bilimoria et al., 2008).

### Grading

Proliferation index, as Ki67 and mitotic count help to assess the ACC prognosis. Very recently, a large European study in localized ACC identified Ki67 as the single most important factor predicting recurrence in patients following R0 resection (Beuschlein et al., 2015). Thus, evaluation of Ki67 indices should be introduced as standard grading in all pathology reports of ACC patients (Beuschlein et al., 2015). More recently, in a large European study on stage IV ACC, the tumor grading, as the association of the Ki67 and the Weiss score, has been considered as an important prognostic parameter of overall survival (Libé et al., 2014), confirming the data on the mitotic count showed in a previous French series (Assie et al., 2007).

#### Molecular markers

Molecular markers issued form genomic and epigenomic analyses are emerging and need to be confronted to the previous mentioned criteria. Hypermethylation status, miRNA profile or driver genes mutations, as TP53, ZNRF3, β-catenin constitute valuable candidates that could integrate a future clinico-molecular prognostic classification of ACC patients (Assié et al., 2014a).

## Treatment

Currently, the only curative approach to ACCs is complete tumor resection. Adjuvant therapies aim to decrease the risk of recurrence. These two approaches address mainly to localized ACCs (stages I, II, some III), also called “ACC amenable to radical resection.” For “unresectable or metastatic ACC” all therapy must be considered palliative, even in some cases (only two tumoral organs, included adrenal), surgery can be considered as an option.

**Figure 1:**
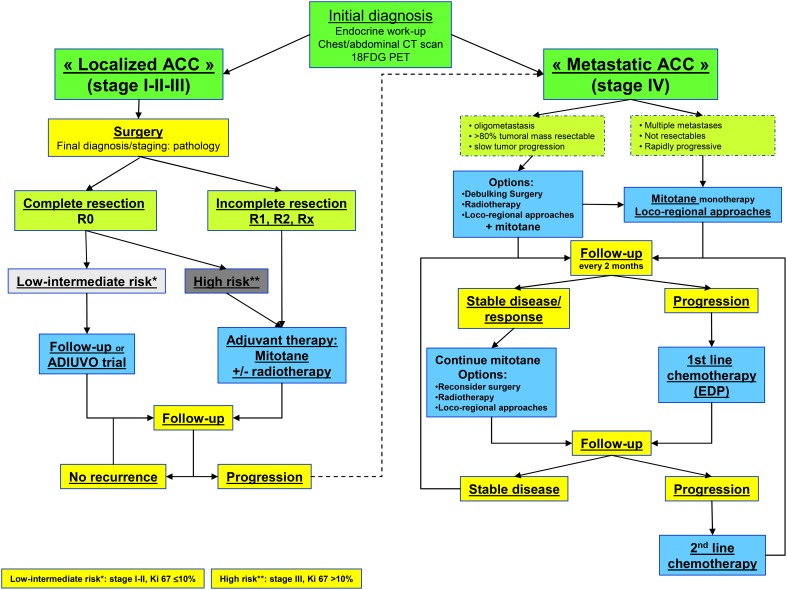
**Flow chart for ACC management**. Abbreviation: R0, complete resection; R1, microscopic incomplete resection; R2, macroscopic incomplete resection; Rx, unknown; EDP, etoposide, doxorubicine, cisplatin.

The Figure 1 showed the current treatment flow-chart for patients with “Localized ACC” and for those with “Metastatic ACC.”

## Localized ACC

### Surgical treatment

For “localized ACC” (“ACC amenable to radical resection”), complete surgical resection (R0) is the treatment of choice. Appropriate preoperative evaluation and operative planning by a surgeon experienced in the resection of ACC (>10 adrenalectomy/year/surgeon) is of the most importance to assure optimal outcome. Different key questions concern the optimal surgical approach: (1) open adrenalectomy (OA) vs. laparoscopic adrenalectomy (LA) (2) lymph nodes dissection (LND) (3) large surgery to the adjacent organs (in bloc surgery).

The choice of the best surgical approach (OA vs. LA) remains controversial. OA should still be regarded as standard treatment for ACC, mainly in the case of an infiltrating tumor or suspected lymph nodes (presumable stage III), and LA should be performed only in selected cases (tumor < 5 cm, absence of higher FDG PET uptake, experimented surgeon).

Up to now, published data comparing the efficacy (and safety) of LA vs. OA for ACC are not definitive, as all the series are retrospectives, with limited number of patients, no follow-up and many biais. Indeed, in two studies has been reported a recurrence rate of 86% in the OA group and 100% in the LA group (with local recurrence and peritoneal carcinomatosis) (Gonzalez et al., 2005; Grubbs et al., 2010). These data has been confirmed by Leboulleux et al. (2010), which showed that peritoneal carcinomatosis occurred in only 25% of patients treated by OA, compared to 60% in LA group. In contrast, other studies reported evidence that LA may be comparable to OA in patients with stage 1 and 2 ACC, in terms of recurrence-free survival. A case-control study from the German ACC Registry Group reported no difference in overall or disease-free survival, tumor capsule violation, or peritoneal carcinomatosis among 117 patients undergoing OA and 35 patients undergoing LA for stage 1–3 ACCs less than 10 cm. However, many patients in the OA group had stage 3 disease and only four patients (11%) undergoing LA were found to have stage 3 disease, potentially introducing a bias toward more advanced disease in the OA group, and 37% of all patients had no data regarding margin status (Brix et al., 2010).

Although no standard management has been established concerning the extent of the first surgery, and in particular the LND, a recent retrospective study, suggests that it might improve both diagnostic accuracy and therapeutic outcome, with a significant reduced risk of tumor recurrence for LND patients (Reibetanz et al., 2011).

As far as, the extension of surgery is concerned, it seems to be little benefit of systematically ipsilateral nephrectomy in the absence of gross local invasion (Gaujoux et al., 2011). However, in order to achieve the complete resection R0, it is mandatory, in case of large tumor and suspicious of organ adjacent invasion or infiltration, to perform a “in bloc” resection, including tumor thrombus embolectomy (Gaujoux and Brennan, 2012).

### Adjuvant therapy

The natural history of recurrence after surgery remains uncertain, but even in case of complete resection, the rate of local recurrence remains important and ranges between 19 and 34%, on the basis of tumor stage. For this reason, adjuvant therapy can be associated after surgery and include mitotane and tumoral bed irradiation.

#### Mitotane

Mitotane is a derivate of the insecticide dichlorodiphenyltrichloroethane (DDD), with adrenolytic and citotoxic activity: in particular, mitotane metabolites inhibit several enzymes in the adrenocortical steroidogenensis pathway, mainly at the level of the cholesterol side-chain cleavage enzymes CYP11A1 and CYP11B1.

In terms of adjuvant therapy in “localized ACC” after surgery, a large retrospective analysis comparing two independent cohorts (Terzolo et al., 2007) demonstrated that patients with adjuvant mitotane had a significantly improved recurrence-free survival. As this is a retrospective study, it remains a matter to discussion. This is particularly true for patients with presumably low or intermediate risk of recurrence (defined by R0 resection, absence of metastases and Ki67 < 10%) (Berruti et al., 2010). For these patients, a prospective international randomized trial (ADIUVO: https://www.epiclin.it/adiuvo) comparing treatment with mitotane vs. a “watch and see” strategy, can be proposed.

#### Mitotane therapy management

The mechanism of mitotane action and pharmacokinetics data are poorly understood. In fact, the variability of individual plasma levels reached by a given dosage is high and it remains unclear which enzyme metabolize mitotane in human, although there is the first evidence that CYP2B6 might be involved. The dose is initiated at 1 g twice daily and increases every 4–7 days by 0.5–1 g/day until a daily dose of 6.0 g/day is reached. Moreover, two different regimens (“high dose” and “low dose”) have been proposed, but no significant difference in mitotane levels and adverse events has been described (Kerkhofs et al., 2010). Several studies demonstrated that a mitotane plasma level ≥14 mg/l is required for clinical efficacy and is associated to a better overall survival (Hermsen et al., 2011). Moreover, the same study demonstrated that even mitotane level > 8 mg/l seems to be associated to a better outcome (Hermsen et al., 2011). Mitotane comes with significant toxicity, like dizziness, vertigo, central nervous system disturbances and gastro-intestinal symptoms. Moreover, as the adrenolytic action of mitotane, all patients develop an adrenal insufficiency, which has to be replaced with a high dosage of hydrocortisone. In fact mitotane induces the cytochrome P450 3A4 (CYP3A4) leading to lower the blood levels of many drugs (including the steroids, anti-hypertensives, antibiotics) (Kroiss et al., 2011a).

#### Radiotherapy

In order to reduce the risk of local recurrence, external radiation therapy of the tumor bed can be an option. In the literature, three retrospective studies with a little number of patients tempt to solve the questions: two of them showed a benefit in preventing local recurrence, but none of them demonstrates an advantage in term of overall survival (Fassnacht et al., 2006; Sabolch et al., 2011; Habra et al., 2013). Currently, an adjuvant therapy is recommended only in case of a particularly high risk for local recurrence (R1 resection) (Berruti et al., 2010).

## Metastatic ACC

In metastatic disease, different parameters had to be considered: the tumoral volume, the number of metastatic organs, the progression slopes. Debulking surgery is only of benefit in patients with a tumoral mass respectable, a limited number of tumoral organs (≤2), with a slight progression and in case of severe hormone excess that cannot be controlled otherwise. Instead, medical therapy should be initiated as soon as the diagnosis is established.

Mitotane remains the only drug approved by the U.S food and drug Administration (FDA) and European Medicine Executive Agency (EMEA) for treatment of “metastatic ACC.” An overview collecting different studies showed that the objective response rate is at best 24% (De Francia et al., 2012).

Recently, the First International Randomized trial in Locally Advanced and metastatic Adrenocortical Carcinoma treatment (FIRM-ACT) trial included 304 patients with metastatic ACC and compared the association of mitotane with etoposide-cisplatin-doxorubicine (M-EDP) with mitotane-streptozotocin (M-Sz) as a first-line or second-line treatment. It was shown that the M-EDP was associated with a better progression-free survival and objective response rate compared to M-Sz (5.0 vs. 2.1 months, 23.2 vs. 9.2%, respectively), although no significant difference was demonstrated on overall survival (Fassnacht et al., 2012). Based on these data, M-EDP is considered as first-line therapy for patients requiring cytotoxic treatment.

For patients failing M-EDP, it has been proposed, as second line chemotherapy, the combination of gemcitabine and capecitabine, leading a disease stabilization for at least 6 months in 29% of patients (Sperone et al., 2009).

### Other treatments

#### Loco-regional approaches

In case of metastatic ACC or ACC recurrence local treatment modalities, such as radiofrequency ablation (RFA) or transarterial chemoembolization (TACE) are recommended.

None of these methods has been explored in clinical trials. However, both methods are an alternative to surgery, when surgery is not desired or contro-indicated, or in order to control the disease locally. RFA has been successfully employed in the palliative setting, rendering patients free of liver metastasis (Ripley et al., 2011). TACE, localized chemoembolization, is based on a selective embolization with injection of a high intratumor levels of cytotoxic substances with a minimum of systemic effects. Predictors of response were a size of <3 cm and high lipidol uptake (Soga et al., 2009).

#### Targeted therapy

Up to now, current treatments fail in many patients with metastatic ACC and different molecular target therapies have been tested. The first trial targeted the epidermal growth factor receptor (EGFR) but the combination of erlotinib and gemcitabine failed to give an objective response (Quinkler et al., 2008). The vascular endothelial growth factor (VEGF) is another potential target, as highly expressed in ACC. In a trial with bevacizumab, a humanized anti-VEGF monoclonal antibody, a progression disease was demonstrated in all of the 10 patients enrolled (Wortmann et al., 2010). Similarly, a multitirosine –kinase inhibitor, sorafenib in combination with paclitaxel did not demonstrate any efficacy in a cohort of 25 patients (Berruti et al., 2012). Only the sunitinib, a multi-TKI, demonstrated in a cohort of 35 patients 14% of stable disease. In this trial concomitant administration of mitotane diminished plasma levels of sunitinib and its active metabolite (Kroiss et al., 2011b).

Recently, drugs targeting IGF-2 seemed to be very promising., as IGF-2 is the most-up regulated gene in ACC. Recently, a phase 2 study used a IMCA12 (cixutumab), a fully humanized IGF-1R antibody showed a lack of efficacy in a cohort of 19 patients (Lerario et al., 2014). In another study, the association of cituximab with temsirolomus, an inhibitor of mammalian targets of IGF-1R signaling, led to a stable disease in 42% of the patients (Naing et al., 2013).

The disappointing results of a huge phase 3 trial “GALACCTIC” with a highly specific IGF-1R inhibitor linstinib (OSI-906) in a cohort of 138 metastatic ACC have been recently published: the progression-free and overall survival did not differ between the “OSI-906” and placebo groups (Fassnacht et al., 2015).

Finally, like in the disease heterogeneity, it appears that using one single agent is not sufficient to induce an objective response. Trials with new targeted substances are under study and alternative combination therapy may be promising.

Recently, as [123I]IMTO single-photon emission CT imaging showed high tracer uptake in issue of adrenocortical origin, [131I]IMTO might represent a suitable compound for targeted radionuclide therapy. [131I]IMTO treatment in 11 patients with advanced ACC resulted in median progression-free survival for 1 month in 6 patients who responded to therapy (Hahner et al., 2012).

### Follow-up

The follow-up management is not well-standardized yet, but, as ACC is an aggressive malignant tumor, patients should be followed every 3 months during and after initial treatment. Only after a recurrence free-time of 2–3 years the surveillance intervals may be increased to 6 months until a completion of follow-up for a total of 5 years. After 5 years of disease-free, the surveillance can be proposed every 1–2 years, because, although rare, some patients can relapse tardily.

Patients should undergo a complete physical examination, hormonal investigations and a complete imaging work-up, including chest and abdominal CT scan. A [18F] FDG-PET may also be considered, even if it's not considered mandatory in the follow-up of ACC patients.

### Conflict of interest statement

The author declares that the research was conducted in the absence of any commercial or financial relationships that could be construed as a potential conflict of interest.

## References

[B1] Arlt W. Biehl M. Taylor A. E. Hahner S. Libe R. Hughes B. A.. (2012). Urine steroid metabolomics as a biomarker tool for detecting malignancy in adrenal tumors. J. Clin. Endocrinol. Metab. 96, 3775–3784. 10.1210/jc.2011-1565 21917861 PMC3232629

[B2] Assie G. Antoni G. Tissier F. Caillou B. Abiven G. Gicquel C.. (2007). Prognostic parameters of metastatic adrenocortical carcinoma. J. Clin. Endocrinol. Metab. 92, 148–154. 10.1210/jc.2006-0706 17062775

[B3] Assié G. Jouinot A. Bertherat J. (2014b). The ‘omics’ of adrenocortical tumours for personalized medicine. Nat. Rev. Endocrinol. 10, 215–228. 10.1038/nrendo.2013.272 24492180

[B4] Assié G. Letouzé E. Fassnacht M. Jouinot A. Luscap W. Barreau O.. (2014a). Integrated genomic characterization of adrenocortical carcinoma. Nat. Genet. 46, 607–612. 10.1038/ng.2953 24747642

[B5] Berruti A. Fassnacht M. Baudin E. Hammer G. Haak H. Leboulleux S.. (2010). Adjuvant therapy in patients with adrenocortical carcinoma: a position of an international panel. J. Clin. Oncol. 28, e401–e402; author reply e403. 10.1200/jco.2009.27.5958 20567001

[B6] Berruti A. Sperone P. Ferrero A. Germano A. Ardito A. Priola A. M.. (2012). Phase II study of weekly paclitaxel and sorafenib as second/third-line therapy in patients with adrenocortical carcinoma. Eur. J. Endocrinol. 166, 451–458. 10.1530/EJE-11-0918 22189997

[B7] Beuschlein F. Weigel J. Saeger W. Kroiss M. Wild V. Daffara F.. (2015). Major prognostic role of ki67 in localized adrenocortical carcinoma after complete resection. J. Clin. Endocrinol. Metab. 100, 841–849. 10.1210/jc.2014-3182 25559399

[B8] Bharwani N. Rockall A. G. Sahdev A. Gueorguiev M. Drake W. Grossman A. B.. (2011). Adrenocortical carcinoma: the range of appearances on CT and MRI. AJR Am. J. Roentgenol. 196, W706–W714. 10.2214/AJR.10.5540 21606258

[B9] Bilimoria K. Y. Shen W. T. Elaraj D. Bentrem D. J. Winchester D. J. Kebebew E.. (2008). Adrenocortical carcinoma in the United States: treatment utilization and prognostic factors. Cancer 113, 3130–3136. 10.1002/cncr.23886 18973179

[B10] Boland G. W. Dwamena B. A. Jagtiani Sangwaiya M. Goehler A. G. Blake M. A. Hahn P. F.. (2011). Characterization of adrenal masses by using FDG PET: a systematic review and meta-analysis of diagnostic test performance. Radiology 259, 117–126. 10.1148/radiol.11100569 21330566

[B11] Brix D. Allolio B. Fenske W. Agha A. Dralle H. Jurowich C. (2010). Laparoscopic versus open adrenalectomy for adrenocortical carcinoma: surgical and oncologic outcome in 152 patients. Eur. Urol. 58, 609–615. 10.1016/j.eururo.2010.06.024 20580485

[B12] De Francia S. Ardito A. Daffara F. Zaggia B. Germano A. Berruti A.. (2012). Mitotane treatment for adrenocortical carcinoma: an overview. Minerva Endocrinol. 37, 9–23. 22382612

[B13] Deandreis D. Leboulleux S. Caramella C. Schlumberger M. Baudin E. (2014). FDG PET in the management of patients with adrenal masses and adrenocortical carcinoma. Horm. Cancer 2, 354–362. 10.1007/s12672-011-0091-5 22076881 PMC10358045

[B14] Duregon E. Volante M. Giorcelli J. Terzolo M. Lalli E. Papotti M. (2009). Diagnostic and prognostic role of steroidogenic factor 1 in adrenocortical carcinoma: a validation study focusing on clinical and pathologic correlates. Hum. Pathol. 44, 822–828. 10.1016/j.humpath.2012.07.025 23158211

[B15] Elsayes K. M. Mukundan G. Narra V. R. Lewis J. S. Jr. Shirkhoda A. Farooki A.. (2004). Adrenal masses: mr imaging features with pathologic correlation. Radiographics 24(Suppl. 1), S73–S86. 10.1148/rg.24si045514 15486251

[B16] Else T. Kim A. C. Sabolch A. Raymond V. M. Kaandathil A. Caoili E. M.. (2014). Adrenocortical carcinoma. Endocr. Rev. 35, 282–326. 10.1210/er.2013-1029 24423978 PMC3963263

[B17] Fassnacht M. Berruti A. Baudin E. Demeure M. J. Gilbert J. Haak H.. (2015). Linsitinib (OSI-906) versus placebo for patients with locally advanced or metastatic adrenocortical carcinoma: a double-blind, randomised, phase 3 study. Lancet Oncol. 16, 426–435. 10.1016/S1470-2045(15)70081-1 25795408

[B18] Fassnacht M. Hahner S. Polat B. Koschker A. C. Kenn W. Flentje M.. (2006). Efficacy of adjuvant radiotherapy of the tumor bed on local recurrence of adrenocortical carcinoma. J. Clin. Endocrinol. Metab. 91, 4501–4504. 10.1210/jc.2006-1007 16895957

[B19] Fassnacht M. Johanssen S. Quinkler M. Bucsky P. Willenberg H. S. Beuschlein F. (2009). Limited prognostic value of the 2004 International Union Against Cancer staging classification for adrenocortical carcinoma: proposal for a Revised TNM Classification. Cancer 115, 243–250. 10.1002/cncr.24030 19025987

[B20] Fassnacht M. Terzolo M. Allolio B. Baudin E. Haak H. Berruti A.. (2012). Combination chemotherapy in advanced adrenocortical carcinoma. N. Engl. J. Med. 366, 2189–2197. 10.1056/NEJMoa1200966 22551107

[B21] Gaujoux S. Al-Ahmadie H. Allen P. J. Gonen M. Shia J. D'Angelica M.. (2011). Resection of adrenocortical carcinoma liver metastasis: is it justified? Ann. Surg. Oncol. 19, 2643–2651. 10.1245/s10434-012-2358-7 22526905

[B22] Gaujoux S. Brennan M. F. (2012). Recommendation for standardized surgical management of primary adrenocortical carcinoma. Surgery 152, 123–132. 10.1016/j.surg.2011.09.030 22306837

[B23] Gonzalez R. J. Shapiro S. Sarlis N. Vassilopoulou-Sellin R. Perrier N. D. Evans D. B.. (2005). Laparoscopic resection of adrenal cortical carcinoma: a cautionary note. Surgery 138, 1078–85. discussion: 1085–1086. 10.1016/j.surg.2005.09.012 16360394

[B24] Groussin L. Bonardel G. Silvera S. Tissier F. Coste J. Abiven G.. (2009). 18F-Fluorodeoxyglucose positron emission tomography for the diagnosis of adrenocortical tumors: a prospective study in 77 operated patients. J. Clin. Endocrinol. Metab. 94, 1713–1722. 10.1210/jc.2008-2302 19190108

[B25] Grubbs E. G. Callender G. G. Xing Y. Perrier N. D. Evans D. B. Phan A. T.. (2010). Recurrence of adrenal cortical carcinoma following resection: surgery alone can achieve results equal to surgery plus mitotane. Ann. Surg. Oncol. 17, 263–270. 10.1245/s10434-009-0716-x 19851811

[B26] Habra M. A. Ejaz S. Feng L. Das P. Deniz F. Grubbs E. G.. (2013). A retrospective cohort analysis of the efficacy of adjuvant radiotherapy after primary surgical resection in patients with adrenocortical carcinoma. J. Clin. Endocrinol. Metab. 98, 192–197. 10.1210/jc.2012-2367 23150683 PMC3537094

[B27] Hahner S. Kreissl M. C. Fassnacht M. Haenscheid H. Knoedler P. Lang K.. (2012). [131I]iodometomidate for targeted radionuclide therapy of advanced adrenocortical carcinoma. J. Clin. Endocrinol. Metab. 97, 914–922. 10.1210/jc.2011-2765 22170726

[B28] Hahner S. Stuermer A. Kreissl M. Reiners C. Fassnacht M. Haenscheid H.. (2008). [123 I]Iodometomidate for molecular imaging of adrenocortical cytochrome P450 family 11B enzymes. J. Clin. Endocrinol. Metab. 93, 2358–2365. 10.1210/jc.2008-0050 18397978

[B29] Hermsen I. G. Fassnacht M. Terzolo M. Houterman S. den Hartigh J. Leboulleux S.. (2011). Plasma concentrations of o,p'DDD, o,p'DDA, and o,p'DDE as predictors of tumor response to mitotane in adrenocortical carcinoma: results of a retrospective ENS@T multicenter study. J. Clin. Endocrinol. Metab. 96, 1844–1851. 10.1210/jc.2010-2676 21470991

[B30] Icard P. Goudet P. Charpenay C. Andreassian B. Carnaille B. Chapuis Y.. (2001). Adrenocortical carcinomas: surgical trends and results of a 253-patient series from the French Association of Endocrine Surgeons study group. World J. Surg. 25, 891–897. 10.1007/s00268-001-0047-y 11572030

[B31] Ilias I. Sahdev A. Reznek R. H. Grossman A. B. Pacak K. (2007). The optimal imaging of adrenal tumours: a comparison of different methods. Endocr. Relat. Cancer 14, 587–599. 10.1677/ERC-07-0045 17914090

[B32] Kebebew E. Reiff E. Duh Q. Y. Clark O. H. McMillan A. (2006). Extent of disease at presentation and outcome for adrenocortical carcinoma: have we made progress? World J. Surg. 30, 872–878. 10.1007/s00268-005-0329-x 16680602

[B33] Kerkhofs T. M. Baudin E. Terzolo M. Allolio B. Chadarevian R. Mueller H. H.. (2010). Comparison of two mitotane starting dose regimens in patients with advanced adrenocortical carcinoma. J. Clin. Endocrinol. Metab. 98, 4759–4767. 10.1210/jc.2013-2281 24057287

[B34] Kerkhofs T. M. Verhoeven R. H. Van der Zwan J. M. Dieleman J. Kerstens M. N. Links T. P.. (2013). Adrenocortical carcinoma: a population-based study on incidence and survival in the Netherlands since 1993. Eur. J. Cancer 49, 2579–2586. 10.1016/j.ejca.2013.02.034 23561851

[B35] Kroiss M. Quinkler M. Lutz W. K. Allolio B. Fassnacht M. (2011a). Drug interactions with mitotane by induction of CYP3A4 metabolism in the clinical management of adrenocortical carcinoma. Clin. Endocrinol. (Oxf) 75, 585–591. 10.1111/j.1365-2265.2011.04214.x 21883349

[B36] Kroiss M. Reuss M. Kuhner D. Johanssen S. Beyer M. Zink M.. (2011b). Sunitinib inhibits cell proliferation and alters steroidogenesis by down-regulation of HSD3B2 in adrenocortical carcinoma cells. Front. Endocrinol. (Lausanne) 2:27. 10.3389/fendo.2011.00027 22654799 PMC3356136

[B37] Leboulleux S. Deandreis D. Al Ghuzlan A. Auperin A. Goere D. Dromain C.. (2010). Adrenocortical carcinoma: is the surgical approach a risk factor of peritoneal carcinomatosis? Eur. J. Endocrinol. 162, 1147–1153. 10.1530/EJE-09-1096 20348273

[B38] Leboulleux S. Dromain C. Bonniaud G. Auperin A. Caillou B. Lumbroso J.. (2006). Diagnostic and prognostic value of 18-fluorodeoxyglucose positron emission tomography in adrenocortical carcinoma: a prospective comparison with computed tomography. J. Clin. Endocrinol. Metab. 91, 920–925. 10.1210/jc.2005-1540 16368753

[B39] Lerario A. M. Worden F. P. Ramm C. A. Hasseltine E. A. Stadler W. M. Else T.. (2014). The combination of insulin-like growth factor receptor 1 (IGF1R) antibody cixutumumab and mitotane as a first-line therapy for patients with recurrent/metastatic adrenocortical carcinoma: a multi-institutional NCI-sponsored trial. Horm. Cancer 5, 232–239. 10.1007/s12672-014-0182-1 24849545 PMC4298824

[B40] Libé R. Borget I. Ronchi C. L. Zaggia B. Kroiss M. Kerkhofs T. (2014). Prognostic factors in Stage III-IV adrenocortical carcinomas (ACC): an European Network for the Study of Adrenal Tumor (ENSAT) study. ASCO Annu. Meet. J. Clin. Oncol. 35:55. 10.1093/annonc/mdv329 26392430

[B41] Lughezzani G. Sun M. Perrotte P. Jeldres C. Alasker A. Isbarn H.. (2010). The European Network for the Study of Adrenal Tumors staging system is prognostically superior to the international union against cancer-staging system: a North American validation. Eur. J. Cancer 46, 713–719. 10.1016/j.ejca.2009.12.007 20044246

[B42] Miller B. S. Gauger P. G. Hammer G. D. Giordano T. J. Doherty G. M. (2010). Proposal for modification of the ENSAT staging system for adrenocortical carcinoma using tumor grade. Langenbecks Arch. Surg. 395, 955–961. 10.1007/s00423-010-0698-y 20694732

[B43] Naing A. Lorusso P. Fu S. Hong D. Chen H. X. Doyle L. A.. (2013). Insulin growth factor receptor (IGF-1R) antibody cixutumumab combined with the mTOR inhibitor temsirolimus in patients with metastatic adrenocortical carcinoma. Br. J. Cancer 108, 826–830. 10.1038/bjc.2013.46 23412108 PMC3590681

[B44] Quinkler M. Hahner S. Wortmann S. Johanssen S. Adam P. Ritter C.. (2008). Treatment of advanced adrenocortical carcinoma with erlotinib plus gemcitabine. J. Clin. Endocrinol. Metab. 93, 2057–2062. 10.1210/jc.2007-2564 18334586

[B45] Reibetanz J. Jurowich C. Erdogan I. Nies C. Rayes N. Dralle H.. (2011). Impact of lymphadenectomy on the oncologic outcome of patients with adrenocortical carcinoma. Ann. Surg. 255, 363–369. 10.1097/SLA.0b013e3182367ac3 22143204

[B46] Ripley R. T. Kemp C. D. Davis J. L. Langan R. C. Royal R. E. Libutti S. K.. (2011). Liver resection and ablation for metastatic adrenocortical carcinoma. Ann. Surg. Oncol. 18, 1972–1979. 10.1245/s10434-011-1564-z 21301973 PMC3272672

[B47] Sabolch A. Feng M. Griffith K. Hammer G. Doherty G. Ben-Josef E. (2011). Adjuvant and definitive radiotherapy for adrenocortical carcinoma. Int. J. Radiat. Oncol. Biol. Phys. 80, 1477–1484. 10.1016/j.ijrobp.2010.04.030 20675074

[B48] Sbiera S. Schmull S. Assie G. Voelker H. U. Kraus L. Beyer M.. (2009). High diagnostic and prognostic value of steroidogenic factor-1 expression in adrenal tumors. J. Clin. Endocrinol. Metab. 95, E161–E171. 10.1210/jc.2010-0653 20660055

[B49] Soga H. Takenaka A. Ooba T. Nakano Y. Miyake H. Takeda M.. (2009). A twelve-year experience with adrenal cortical carcinoma in a single institution: long-term survival after surgical treatment and transcatheter arterial embolization. Urol. Int. 82, 222–226. 10.1159/000200804 19322014

[B50] Sperone P. Ferrero A. Daffara F. Priola A. Zaggia B. Volante M.. (2009). Gemcitabine plus metronomic 5-fluorouracil or capecitabine as a second-/third-line chemotherapy in advanced adrenocortical carcinoma: a multicenter phase II study. Endocr. Relat. Cancer 17, 445–453. 10.1677/ERC-09-0281 20410174

[B51] Sturgeon C. Shen W. T. Clark O. H. Duh Q. Y. Kebebew E. (2006). Risk assessment in 457 adrenal cortical carcinomas: how much does tumor size predict the likelihood of malignancy? J. Am. Coll. Surg. 202, 423–430. 10.1016/j.jamcollsurg.2005.11.005 16500246

[B52] Terzolo M. Angeli A. Fassnacht M. Daffara F. Tauchmanova L. Conton P. A.. (2007). Adjuvant mitotane treatment for adrenocortical carcinoma. N. Engl. J. Med. 356, 2372–2380. 10.1056/NEJMoa063360 17554118

[B53] Tissier F. Aubert S. Leteurtre E. Al Ghuzlan A. Patey M. Decaussin M.. (2012). Adrenocortical tumors: improving the practice of the Weiss system through virtual microscopy: a National Program of the French Network INCa-COMETE. Am. J. Surg. Pathol. 36, 1194–1201. 10.1097/PAS.0b013e31825a6308 22790860

[B54] Weiss L. M. (1984). Comparative histologic study of 43 metastasizing and nonmetastasizing adrenocortical tumors. Am. J. Surg. Pathol. 8, 163–169. 10.1097/00000478-198403000-00001 6703192

[B55] Wortmann S. Quinkler M. Ritter C. Kroiss M. Johanssen S. Hahner S.. (2010). Bevacizumab plus capecitabine as a salvage therapy in advanced adrenocortical carcinoma. Eur. J. Endocrinol. 162, 349–356. 10.1530/EJE-09-0804 19903796

[B56] Young W. F. Jr. (2011). Conventional imaging in adrenocortical carcinoma: update and perspectives. Horm. Cancer 2, 341–347. 10.1007/s12672-011-0089-z 21997291 PMC10358061

[B57] Zhang H. M. Perrier N. D. Grubbs E. G. Sircar K. Ye Z. X. Lee J. E.. (2010). CT features and quantification of the characteristics of adrenocortical carcinomas on unenhanced and contrast-enhanced studies. Clin. Radiol. 67, 38–46. 10.1016/j.crad.2011.03.023 21783181 PMC3889671

